# Identification of an apoptosis-related prognostic gene signature and molecular subtypes of clear cell renal cell carcinoma (ccRCC)

**DOI:** 10.7150/jca.51812

**Published:** 2021-04-02

**Authors:** Weimin Zhong, Fengling Zhang, Chaoqun Huang, Yao Lin, Jiyi Huang

**Affiliations:** 1 The Fifth Hospital of Xiamen, Xiamen 361101, Fujian Province, China.; 2 Key Laboratory of Optoelectronic Science and Technology for Medicine of Ministry of Education, College of Life Sciences, Fujian Normal University, Fuzhou 350117, Fujian Province, China.

**Keywords:** clear cell renal cell carcinoma, apoptosis, gene signature, nomogram, molecular subtypes, drug sensitivity, immunotherapy.

## Abstract

Previously studies have shown that apoptosis-related genes play an essential role in normal cell turnover, maintaining the immune system function, and inducing cell death. However, their prognostic roles in clear cell renal cell carcinoma (ccRCC) have not been thoroughly investigated. In the present study, apoptosis-related genes expression profiles from The Cancer Genome Atlas (TCGA) and International Cancer Genome Consortium (ICGC) database were used as training dataset and external validation dataset, respectively. According to the systematical analysis of the apoptosis-related gene expression profile, we constructed a gene signature to determine the role of apoptosis-related genes in the survival of ccRCC. We discovered that patients in the low-risk group have a better survival than high-risk group and the signature could serve as an independent prognostic factor. A nomogram, including a signature and clinical factors, were constructed to estimate the individual survival probability. The Gene set enrichment analysis (GSEA) identified some significant pathways which may contribute to understanding the underlying mechanism of ccRCC. In addition, the prognostic efficiency of the risk model was further validated in the disease free survival (DFS) and the ICGC dataset, respectively. We also identified three molecular subtypes (named C1, C2, and C3) based on apoptosis-related gene expression. We found that C1 was corresponding to a worse survival outcome and showed a high drug sensitivity of sorafenib and sunitinib. C2 and C3 were corresponding to a better survival outcome and presented a low drug sensitivity to sorafenib and sunitinib. Moreover, we found that C2 and C3 have more likelihood to be respond to immunotherapy. Together, the apoptosis-related gene signature and three molecular subtypes may promote the understanding of the underlying molecular mechanism of ccRCC, and provided reference for developing individualized treatment of the ccRCC patients.

## Introduction

Worldwide, renal cell carcinoma (RCC) is one of the most common types of cancers, which is responsible for 2-3% cases of all adult malignant tumors. Statistically, as the high heterogeneous tumor, ~270, 000 new cases are diagnosed per year 1. According to the cytogenetic and histological features, RCC can be mainly classified into three subtypes including clear cell renal cell carcinoma (ccRCC), papillary renal cell carcinoma (pRCC) and chromophobe renal cell carcinoma (chRCC) type, of which ccRCC account for 70-80% RCC cases 2.

Different from other RCC subtype malignancies, ccRCC shows resistance to conventional chemotherapy and radiotherapy, especially for the advanced ccRCC, which promotes the development of alternative therapies, such as targeted therapy and immunotherapy. Currently, numerous promising immunotherapy drugs, including PD-1/PD-L1, interleukin-2 (IL-2), and interferon (IFN) blocking agents, have been approved for the treatment of ccRCC 3, and the overall therapeutic effect is satisfactory 4. However, some patients still showed poor responses and developed to drug resistance 5. Moreover, targeted therapy drugs, including sunitinib and sorafenib, have been approved for the treatment of metastatic RCC, with fewer side effects and better selectivity than immunotherapy 6, 7. Despite these progresses, patients with ccRCC still showed drug resistance 2, 8. Consequently, there is an urgent need to identify more effective biomarkers and novel therapeutic targets for the treatment of ccRCC.

Apoptosis also called programmed cell death is a primary cellular mechanism for mammals to eliminated DNA-damage cells and maintained tissue homeostasis 9, 10. There are two main pathways, including the extrinsic pathway and the intrinsic pathway, to activate apoptosis 11. The tumor cells can evade apoptosis via many ways. For example, the up-regulation of anti-apoptotic BCL-2 proteins and loss of BAX and/or BAK can inhibit the apoptosis function and promote tumorigenesis 12. Moreover, inhibiting caspase function also can prevent the apoptosis function 12. The loss of apoptosis will increase tumor cells survival time and accumulate the mutations, which can enhance invasiveness during tumor cell progression, stimulate the angiogenesis of tumors, and promote cell proliferation 13.

In the present study, we aimed to systemically analyze the expression of apoptosis-related genes listed in the TCGA dataset and ICGC dataset. We developed and validated an apoptosis-related gene signature and demonstrated that it could serve as an independent prognostic biomarker in ccRCC. Also, we identified three molecular subtypes named C1, C2, and C3. The subtypes exhibit distinct drug sensitivity to the sunitinib and sorafenib and probability to the immunotherapy.

## Materials and Methods

### Data collection

Firstly, a total of 630 ccRCC samples' expression profiles and corresponding clinical information were downloaded from The Cancer Genome Atlas database (TCGA, https://portal.gdc.cancer.gov/, N= 539) and the International Cancer Genome Consortium database (ICGC, https://icgc.org/, N=91), respecttively. In order to ensure a reliability of the survival result in the TCGA dataset, we excluded the patients with survival time less 30 days and incomplete clinical information (survival status, survival time, age, gender, smoking, stage, and grade). As a result, 512 samples from TCGA with survival time ≥ 30 days and complete clinical information (survival status, survival time, age, gender, smoking, stage, and grade) were served as the training dataset. The similar screening criterion was also performed on the ICGC dataset, and finally 90 samples were included our analysis as the external validation dataset. Moreover, we also obtained 161 apoptosis-related genes from the Molecular Signatures Database (MSigDB V7.1, https://www.gsea-msigdb.org/gsea/msigdb/index.jsp).

### Risk model construction

The univariate cox regression analysis was conducted on apoptosis-related genes in the TCGA dataset using the “survival” R package. To increase the reliability and feasibility of the clinical prognosis of genes, we then made a selection based on genes that screened from univariate cox regression analysis result with *p*-value ≤ 0.05. Robust likelihood-based survival (rbsurv) analysis was performed using the “rbsurv” R package 14. The genes found to be significant from the result of robust likelihood-based survival analysis were further applied to multivariate stepwise cox regression analysis to obtain the coefficient. According to the coefficient, the risk formula was built as:


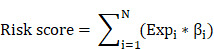


where Exp _i_ represents each gene expression and β _i_ represents the coefficient of each gene.

### Survival analysis

The patients were categorized into the low-risk group and the high-risk group based on the median risk score. The survival difference between groups was identified using the kaplan-meier and log-rank test analysis in a “survminer” R package (https://cran.r-project.org/web/packages/survminer). The univariate cox regression analysis and multivariate cox regression analysis were performed to determine the signature risk score as an independent prognostic factor. The receiver operating characteristic (ROC) curves analysis was used to evaluate the sensitivity and specificity of gene signature in “survivalROC” (https://cran.r-project.org/web/packages/survivalROC) R package. The ICGC dataset was served as the validation dataset to confirm the predictive capability of the gene signature. Moreover, the nomogram and calibration plots analysis were conducted on the risk score and clinical traits (grade, stage, and age) in the TCGA dataset by using the “rms” R package.

### Gene Set Enrichment Analysis (GSEA) and Gene Set Variation Analysis (GSVA)

The GSEA analysis was performed to explore the association between the gene signature risk score and pathway. The “c2.cp.kegg.v7.1.symbols.gmt” file was selected as a reference gene set, and the permutations were performed 1,000 times for each analysis. Moreover, the GSVA analysis was performed to calculate the scores for each ccRCC patients based on the defined gene sets of pathways. The significant pathways were screened with the FDR < 0.05.

### Characterization of molecular subtypes of ccRCC

The previously downloaded apoptosis-related genes were exploited to non-negative matrix factorization (NMF) clustering analysis 15. Before NMF clustering, a filtering step was performed. We retained the top 100 variance features genes in TCGA and ICGC dataset. The NMF clustering was further performed on these genes using the "NMF" R package, and the optimal k value was selected when the cophenetic correlation coefficient began to decline 16. Moreover, principal components analysis (PCA) was employed to estimate the classification effect using the R package "princomp". The Tumor Immune Dysfunction and Exclusion (TIDE, http://tide.dfci.harvard.edu/) algorithm and subclass mapping (SubMap) analysis were applied to predict the clinical response to immunotherapy and immune checkpoint blockade.

### The prediction of chemotherapeutic response

The chemotherapeutic response of each sample in TGCA was predicted on the Genomics of Drug Sensitivity in Cancer (GDSC, https://www.cancerrxgene.org/) database. Two commonly used drugs, Sorafenib and Sunitinib, which have been approved for the treatment of RCC, were selected. The prediction procedure was performed by using the "pRRophetic" R package, and the half-maximal inhibitory concentration (IC_50_) was evaluated using the ridge regression analysis 17. Prediction accuracy was assessed by 10-fold cross-validation based on the GDSC training set.

### Statistical Analysis

All analysis was implemented in the R 3.6.2 environment. The categorical data were used for the Fisher's exact test or chi-square test, while the continuous data were used for the Kruskal-Wallis test. For all statistical analyses, a *P* value ≤ 0.05 was regarded as statistically significant.

## Results

### Acquisition of apoptosis-related gene

A total of 161 apoptosis-related genes were retrieved from the molecular signature database v7.1 (MsigDB, https://www.gsea-msigdb.org/gsea/msigdb). The expression profile of these genes was further obtained from TCGA and ICGC datasets, respectively. In total, 512 patients in TCGA and 90 patients in ICGC with their corresponding clinical information were exploited in the downstream analysis, among them the TCGA dataset was exploited as the training dataset and ICGC was the external validation dataset (Table 1 and Table 2).

### Construction of apoptosis-related gene signature

**Figure 1:**
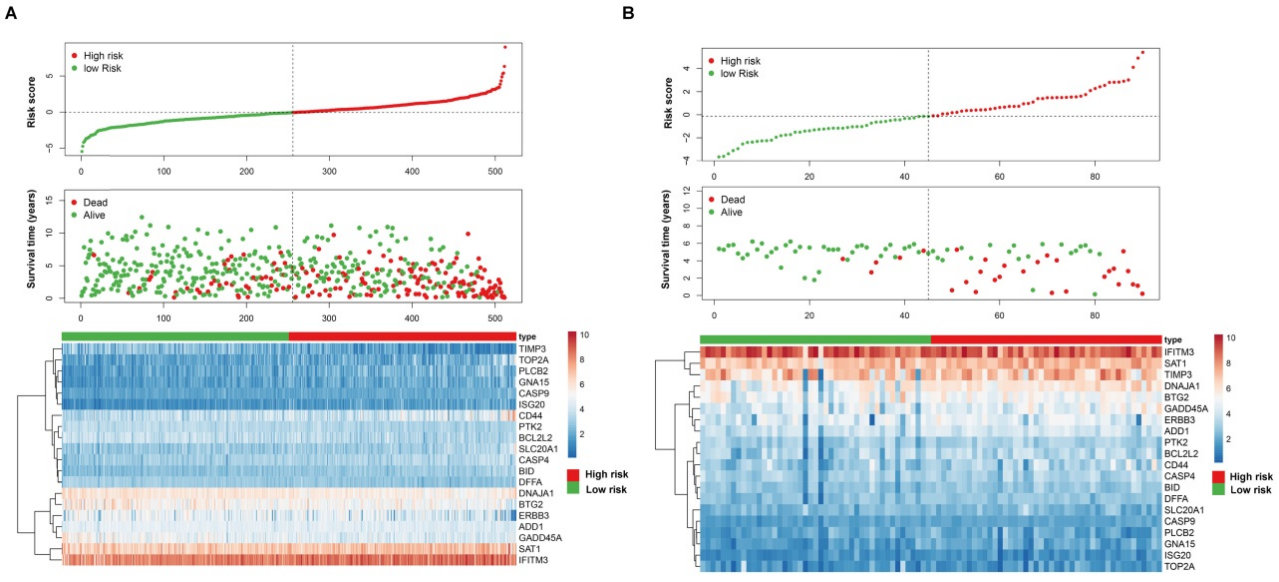
The apoptosis-related gene signature risk score analysis in the TCGA dataset (A) and ICGC dataset (B). The upper panel represent represent the risk distribution; middle panel represent the survival time (years) of ccRCC patients that ranked by the risk parameters in a descending order, lower panel showed the apoptosis-related gene expression for each patients.

**Figure 2:**
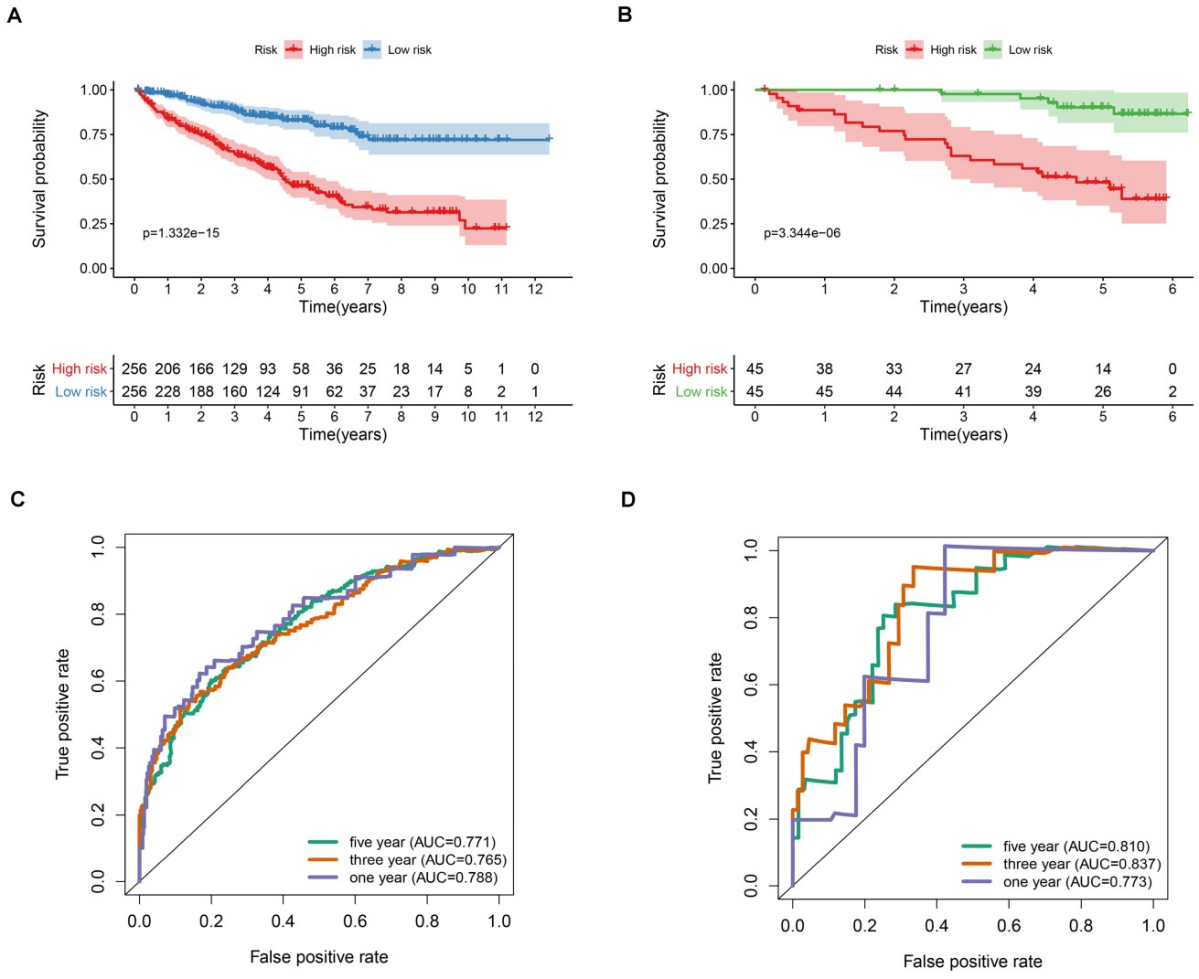
Prognostic value of the apoptosis-related gene signature in ccRCC. (A) Kaplan-Meier overall survival curves analysis for the patients assigned to high-risk and low-risk group in TCGA dataset. (B) Kaplan-Meier overall survival curves analysis for the patients assigned to high-risk and low-risk group in ICGC dataset. (C) Receiver operating characteristic (ROC) analysis of the accuracy for the apoptosis-related gene signature based risk score in the TCGA dataset. (D) Receiver operating characteristic (ROC) analysis of the accuracy for the apoptosis-related gene signature based risk score in the ICGC dataset.

After screening the genes shared in the two datasets, a total of 150 apoptosis-related genes were analyzed using univariate cox regression analysis, and a total of 81 significant genes were selected (p < 0.05). We further used the robust likelihood-based survival analysis (rbsurv) to make a selection for target genes. We then exploited it in the multivariate stepwise cox regression analysis. As a result, 20 significantly apoptosis-related genes were selected (Table S1). According to the cox regression coefficient, we established a 20-gene signature. We then calculated the risk score for each patient in the TCGA dataset and ICGC dataset based on risk formula. Thus, patients were divided into the high-risk and the low-risk group, respectively. As shown in Figure 1A and B, patients in the high-risk score were corresponding to more death cases, while patients with prolonged survival time tend to have a low-risk score. The kaplan-meier (K-M) curve and log-rank test results indicated that patients in the low-risk group and high-risk group have a significant survival difference in the TCGA dataset and ICGC dataset (p < 0.001), respectively (Figure 2A-B). The receiver operating characteristic curve (ROC) results demonstrated that a gene signature has a good performance in prediction for the survival of ccRCC (Figure 2C-D). Despite the prognostic efficiency of the risk model was demonstrated in the ICGC validation cohort. Considering the importance of the disease free survival info, we further evaluated the accuracy of the risk model. As showed in Figure S1A, the K-M curve analysis result identified that a significant survival divergence between high-risk and low-risk group (p < 0.001). The ROC analysis result indicated that the accuracy of the mode in 1-, 3-, and 5-year were 0.753, 0.735, and 0.722, respectively (Figure S1B). In addition, we also demonstrated that the risk model can serve as an independent prognostic factor for the DFS in ccRCC through the univariate cox regression analysis (Figure S1C) and multivariate cox regression analysis (Figure S1D).

### Association between clinical traits and apoptosis-related gene signature

**Figure 3:**
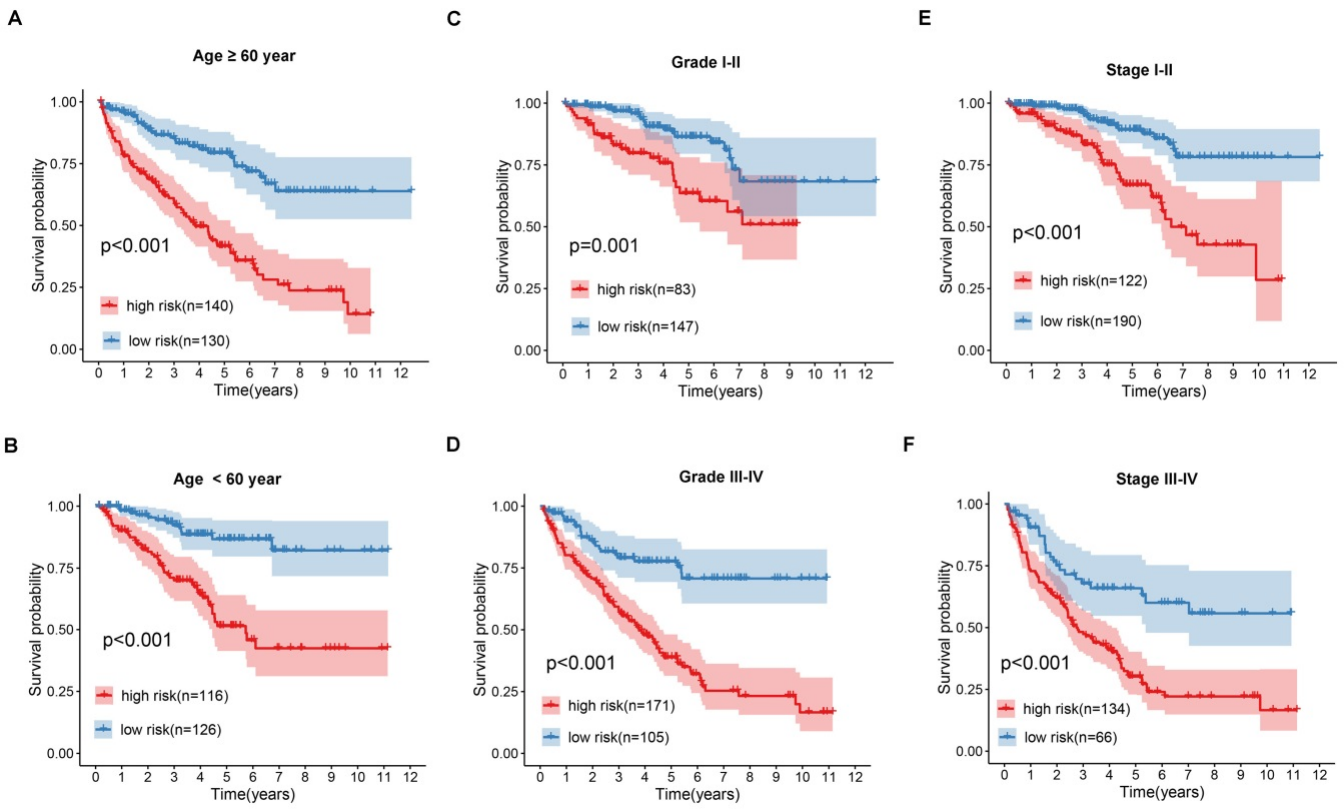
Kaplan-Meier (K-M) survival analysis of the signature risk score in ccRCC patients stratified by age (A and B), grade (C and D) and pathological stage (E and F).

**Figure 4:**
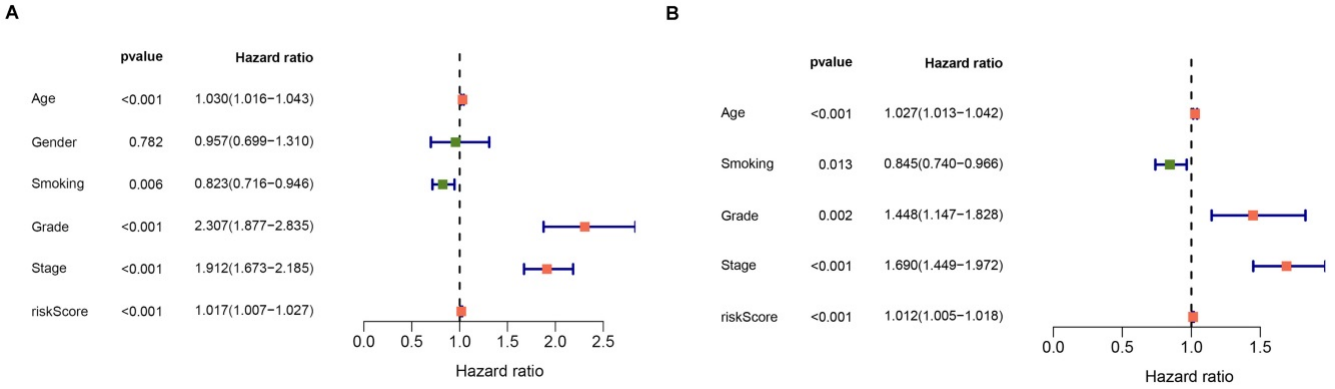
Forest plot of the univariate and multivariate cox regression analyses evaluating the independent prognostic value of apoptosis signature in ccRCC patients.

The prognostic value of the apoptosis-gene signature in the ccRCC clinical features was investigated by performing the K-M curve analysis and log-rank test. As shown in Figure 3A-F, the apoptosis-gene signature exhibited a significant prognostic value in ccRCC patients stratified by age, grade, and stage, suggesting that the apoptosis-related gene signature can predict the overall survival of ccRCC without considering the clinical factors. Moreover, the univariate cox regression analysis and multivariate cox regression analysis were used to evaluate the independent prognostic value of apoptosis-related gene signature in the prognosis of ccRCC. As shown in Figure 4A and 4B, the risk score of the stage, grade, and age was listed as the independent risk factor associated with poor overall survival of ccRCC. Besides, we discovered that the risk score was significantly increased in stage and grade while showed no apparent difference in age (Figure S2A-C). These results indicated that apoptosis-related gene signature might have a significant impact on the malignant progression of ccRCC.

### Gene set enrichment analysis for the apoptosis-related gene signature

**Figure 5:**
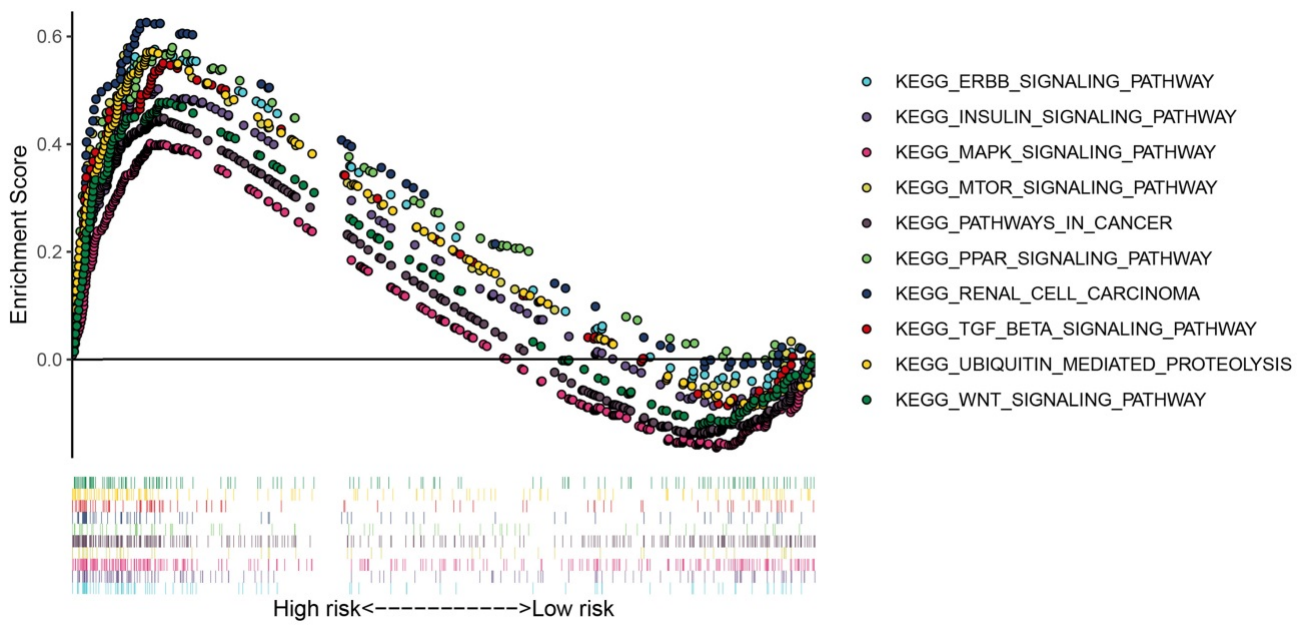
GSEA enrichment analysis for the low risk group of the apoptosis-related gene signature.

To further explore the potential pathways of the apoptosis risk group in the ccRCC, we performed gene set enrichment analysis between the high-risk group and the low-risk group. As shown in Figure 5, patients in the low-risk group mainly involved in the ERBB signaling pathway, MAPK signaling pathway, MTOR signaling pathway, WNT signaling pathway, insulin signaling pathway, and renal cell carcinoma pathway, etc.

### Construction of nomogram based on gene signature and risk factors

**Figure 6:**
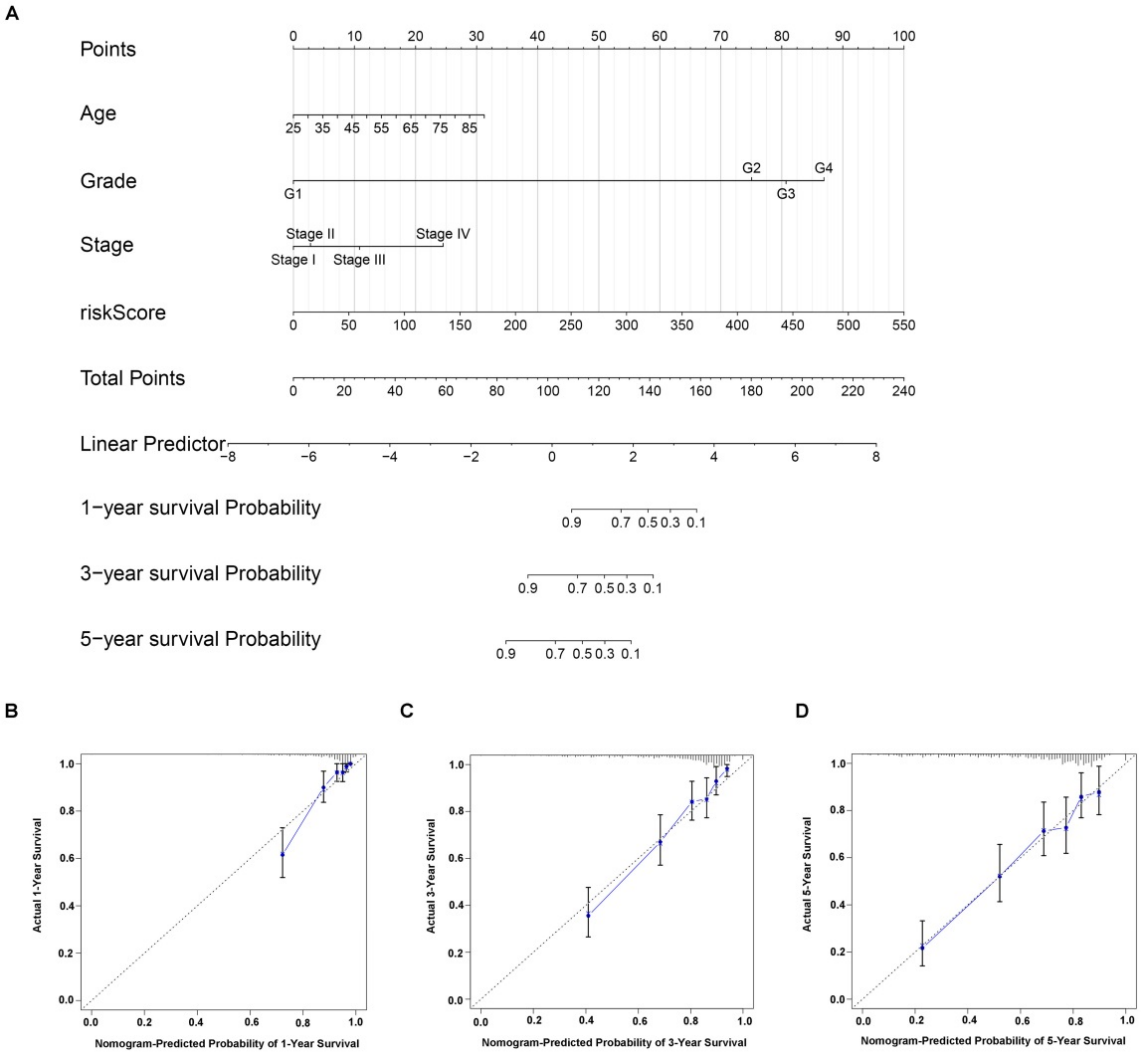
Construction of individualized prediction model for the survival of ccRCC patients. (A) A nomogram prediction model that developed on the basis of risk score, age, stage, grade for the overall survival of ccRCC patients in 1-, 3- and 5-year. Calibration curves validation of nomogram for predicting overall survival in 1-year (B), 3-year (C) and 5-year (D) of ccRCC patients.

Nomogram is a powerful tool used to estimate the prognosis of oncology and medicine. By integrating gene signature risk model and independent risk factors (age, stage, and grade), we built a nomogram for ccRCC (Figure 6A). The C-index value was 0.774, indicating the high accuracy of the nomogram. Moreover, the calibration curve result displayed a high consistency in the probability of 1-, 3- and 5-year overall survival between the actual observation and the nomogram prediction (Figure 6C-D).

### Association between the apoptosis-related gene signature and tumor immune microenvironment

**Figure 7:**
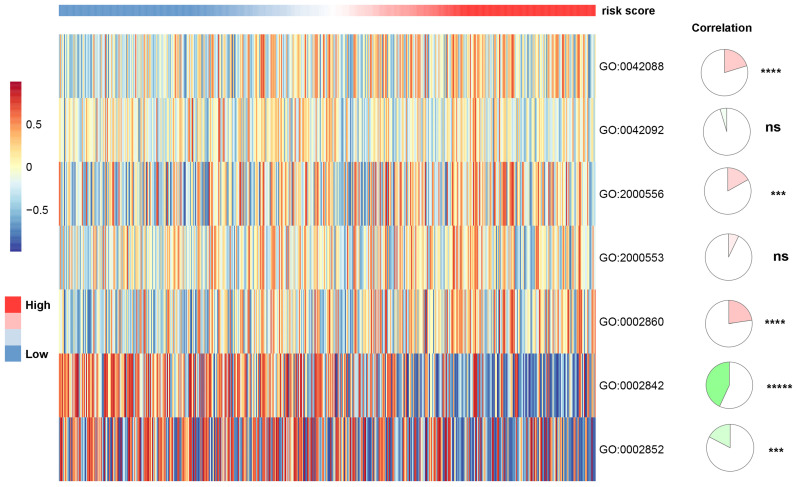
The association between T-cell-related immunity and apoptosis-related gene signature. T-helper 1 type immune response (GO:0042088); T-helper 2 type immune response (GO:0042092); positive regulation of T-helper 1 cell cytokine production (GO:2000556); positive regulation of T-helper 2 cell cytokine production (GO:2000553); positive regulation of natural killer cell-mediated cytotoxicity directed against tumor cell target (GO:0002860); positive regulation of T cell-mediated immune response to tumor cell (GO:0002842); regulation of T cell-mediated cytotoxicity directed against tumor cell target (GO:0002852).

To investigate the association between apoptosis-related gene signature and immune infiltration level, we used the CIBERSORTx online tools to calculate the infiltration level of 22 immune cells based on the gene expression data. As shown in Figure S3, the gene signature risk score was positively correlated with T cells CD4 memory activated (spearman coefficient = 0.46, *p* vale < 0.0001). We further applied the gene set variation analyses (GSVA) to explore the association between apoptosis-related gene signature and T-cell immune response in ccRCC. We discovered that the apoptosis-related gene signature was positively correlated T-helper 1 type immune response, positive regulation of T-helper 1 type cytokine production. Also, it presented a negative correlation with positive regulation of T cell-mediated immune response to the tumor cell and regulation of T cell-mediated cytotoxicity directed against tumor cell target, indicating that apoptosis plays a role in T-cell immunity to tumors (Figure 7).

### Identification of molecular subtypes of ccRCC

**Figure 8:**
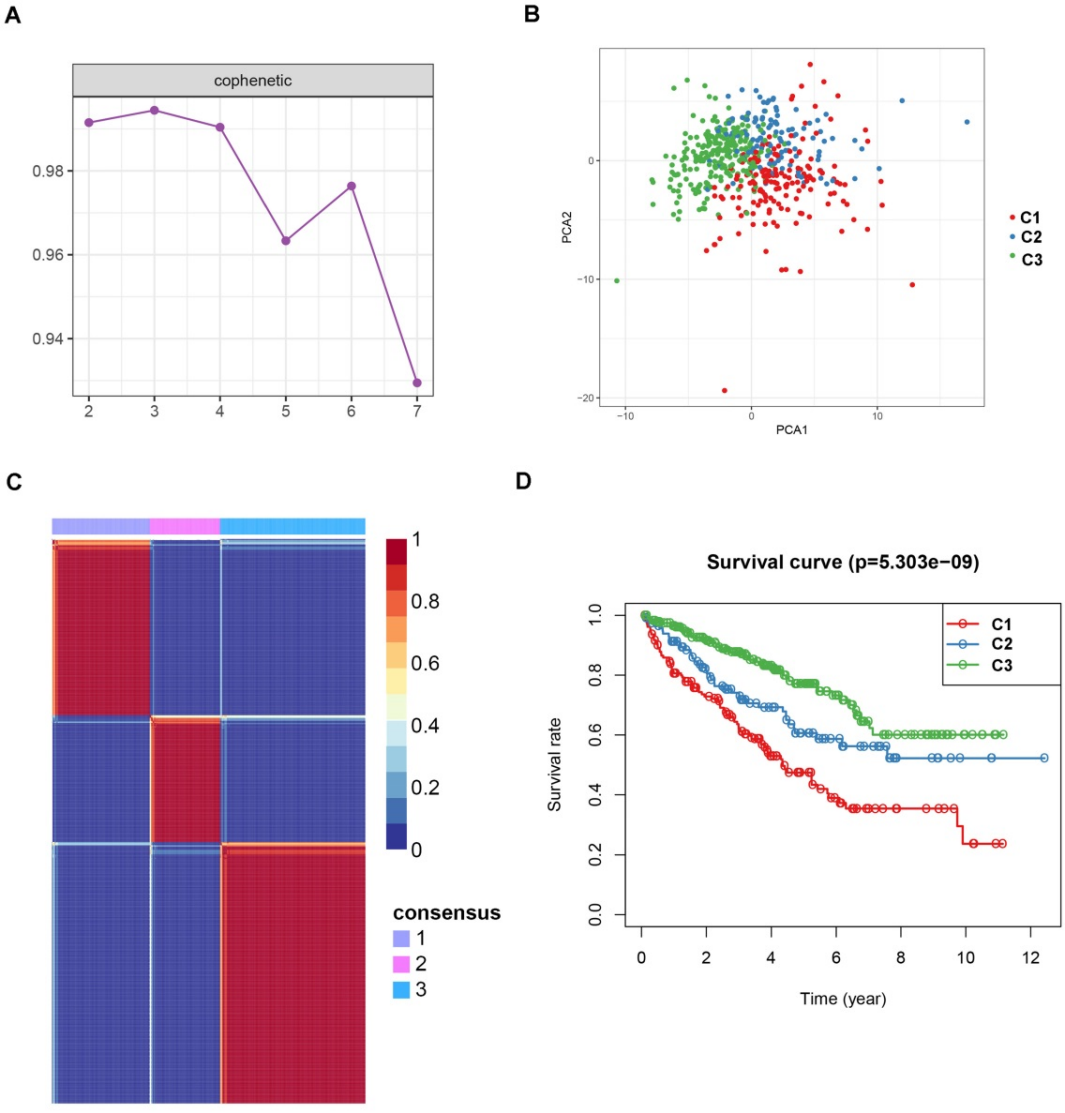
Non-negative matrix factorization (NMF) clustering analyses for apoptosis-related genes in TCGA dataset. (A) The cophenetic correlation coefficient was calculated when k = 2 to k = 7. (B) Principal components analysis for the apoptosis-related genes, each dots represent a sample. (C) Non-negative matrix factorization clustering heatmap for apoptosis-related genes when k =3. (D) Kaplan-Meier survival analysis for the ccRCC patients when k =3.

**Figure 9:**
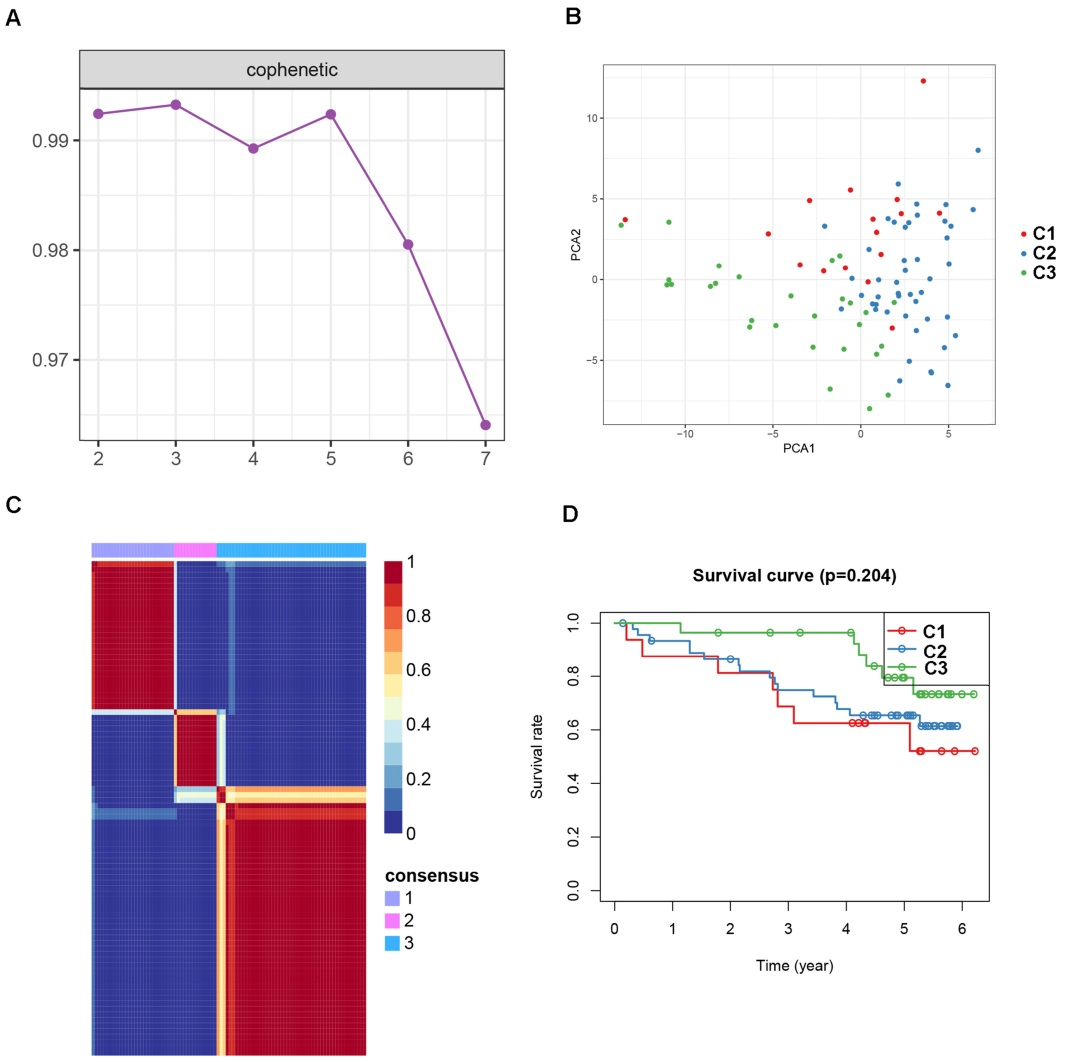
Non-negative matrix factorization (NMF) clustering analyses for apoptosis-related genes in ICGC dataset. (A) The cophenetic correlation coefficient was calculated when k = 2 to k = 7. (B) Principal components analysis for the apoptosis-related genes, each dots represent a sample. (C) Non-negative matrix factorization clustering heatmap for apoptosis-related genes when k =3. (D) Kaplan-Meier survival analysis for the ccRCC patients when k =3.

**Figure 10:**
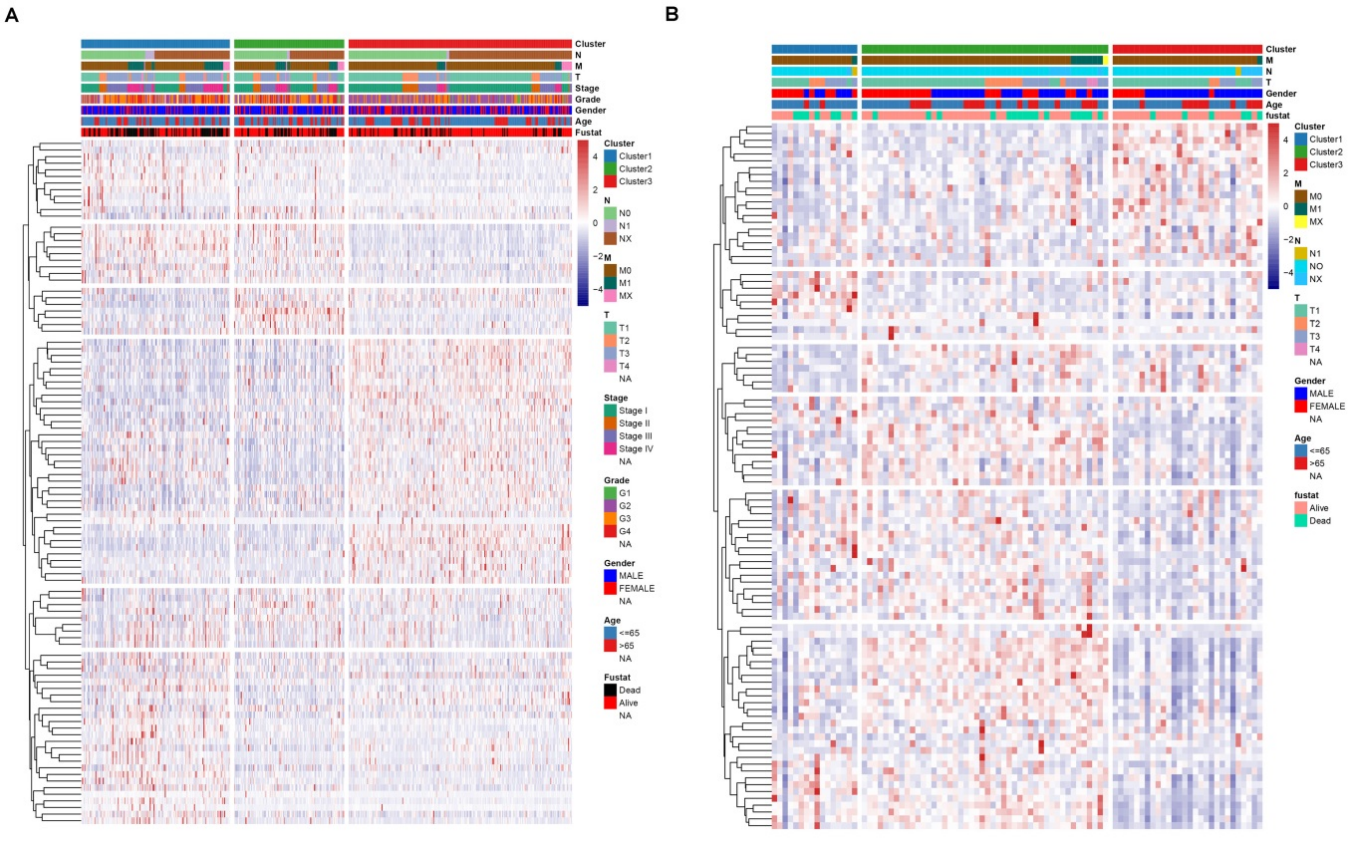
Association between subtypre and clinical feature in TCGA dataset (A) and ICGC dataset (B).

To identify potential molecular subtypes of ccRCC, the previously downloaded apoptosis-related genes were selected as the NMF cluster analysis. The cophenetic correlation coefficients were calculated to determine the optimal k value, and k =3 was chosen as the optimal cluster number after a comprehensive consideration (Figure 8A, cluster names C1, C2, and C3). The principal components analysis (PCA) analysis result and cluster heatmap showed a clear difference when k =3, indicating the robust and reliable clustering of the samples (Figure 8B-C). Other subtype survival analysis result revealed that C3 was corresponding to a better survival outcome while C1 associated with worse survival (*P* = 5.303e-09) (Figure 8D). Also, a similar cluster result was validated in the ICGC dataset. However, the subtype survival result was not significant, possibly due to the small number of sample size (*P*-value = 0.204) (Figure 9). We also found that some genes were differentially expressed between subtypes (Figure 10).

### Correlation of ccRCC subgroups with mutation and immune checkpoint

**Figure 11:**
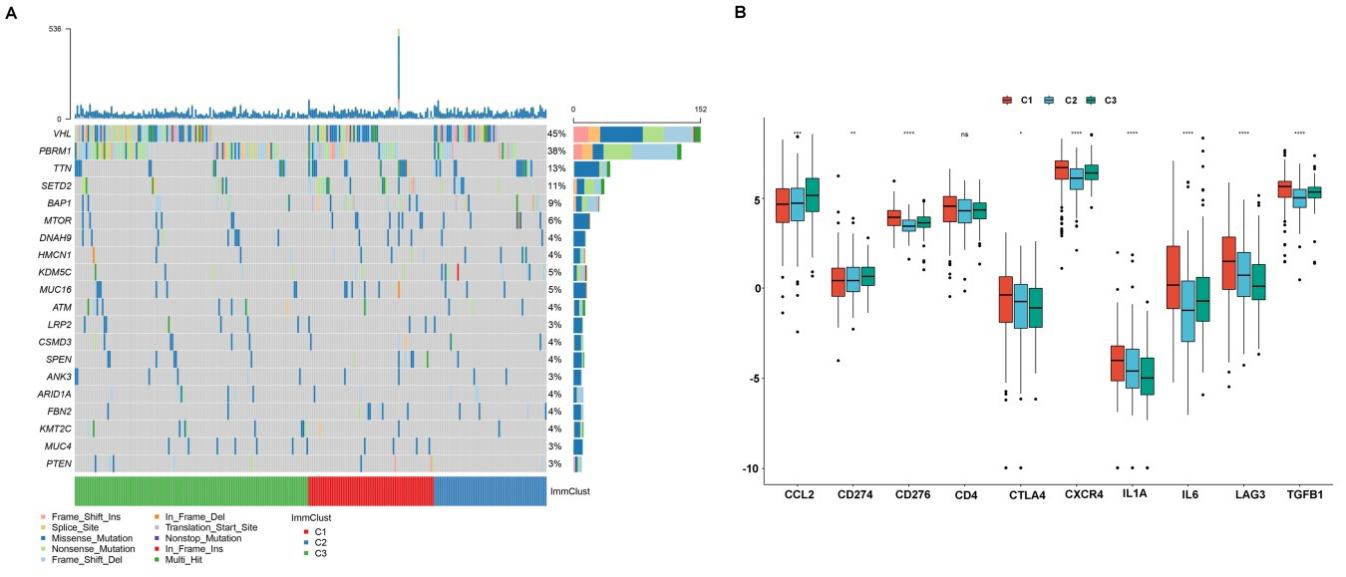
The relationship between ccRCC subtypes and mutations and immune check point. (A) Oncoprint of mutation frequencies for ccRCC subtypes. (B) the expression profile of known immune checkpoint of ccRCC subtypes.

Cumulative evidence showed that the tumoral genomic landscape was tightly associated with anti-tumor immunity. To explore the difference in the somatic mutation frequency among three subgroups, we retrieved the somatic mutation data from the TCGA database. As shown in Figure 11A, VHL is the most common mutation gene in ccRCC, and we observed that subtype C3 was corresponding to the highest mutations, while subtype C1 and subtype C2 were inclined to middle and lowest mutation frequencies. Besides, we investigate the relationship between subtypes and expression levels of immune checkpoint genes that were selected based on current drug inhibitors or have been approved for the treatment of cancers. We found that the expression level of CCL2 and CD274 (PD-L1) was significantly increased from subtype C1 to C3. The expression level of CTLA4, IL1A, LAG3 presented a decreasing trend from C1 to C3. The CD276 (B7-H3), CXCR4, IL6, and TGFB1 were showed a high expression level in C1 and exhibited medium and lower expression levels in C3 and C2 (Figure 11B).

### Immuno/Chemotherapies for ccRCC subtype

**Figure 12:**
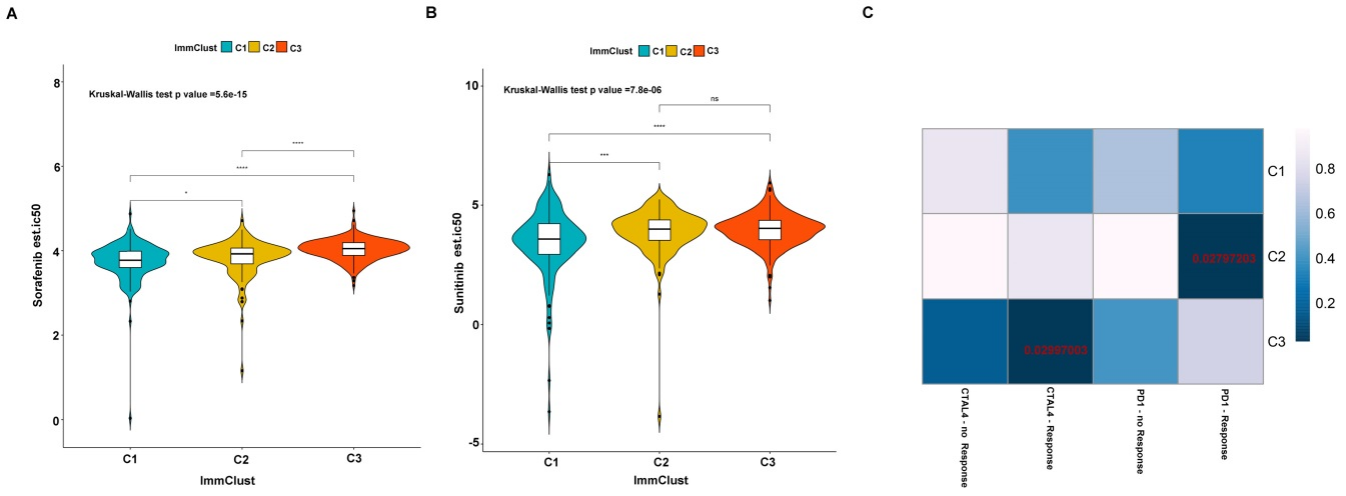
Differential putative chemotherapeutic and immunotherapeutic response for the ccRCC patients. The violine box plots of estimated IC50 for Sorafenib (A) and Sunitinib (B) in subtypes of ccRCC. (C) Submap analysis revealed that subtype C2 is more likely responded to programmed cell death protein 1 inhibitor (Bonferroni-corrected P value = .0280) and subtype C3 is more sensitive to cytotoxic T-lymphocyte-associated protein 4 inhibitor (Bonferroni-corrected P value = .0300).

Previously studies have reported that sorafenib and sunitinib were applied to the treatment of metastatic RCC in 2005 and 2006, respectively. Thus, we further evaluate the response of the three subtypes to the two drugs. We use ridge regression to train a prediction model on the GDSC cell line dataset and evaluate the satisfactory prediction accuracy through 10-fold cross-validation. We calculated the half-maximal inhibitory concentration (IC_50_) value for each sample in the TCGA dataset based on the predictive model of the two drugs. We found that a significant divergence in the IC_50_ between three groups and subtype C1 is more sensitive to the two drugs (Kruskal-Wallis *P*-value =5.6e-15 for sorafenib and Kruskal-Wallis *P*-value =7.8e-06 for sunitinib) (Figure 12A-B). Presently, although the immunotherapy drugs, including PD-1/PD-L1 blocking agents, have been used to ccRCC treatment, some patients remain responded poorly. We therefore applied the TIDE algorithm to predict the probability of response to immunotherapy, and the result indicated that subtype C2 (42/117 = 0.359) and subtype C3 (65/237 = 0.274) are more likely to respond to immunotherapy compared to subtype C1 (31/158 = 0.196). We also employed the submap tools to compare the expression profile of the three subtypes with a published melanoma dataset, which contained 47 patients that responded to immunotherapies. We discovered that subtype C2 is more sensitive to respond to anti-PD-1 therapy, and subtype C3 is more susceptible to anti-CTAL4 treatment (Figure 12C).

## Discussion

ccRCC is the most frequent subtype of RCC, which has a poor prognosis and lack of effective markers. In the current study, we collected the expression of apoptosis-related genes and corresponding clinical information from the TCGA dataset and ICGC dataset. By performing a series of bioinformatic analyses (univariate and multivariate cox regression analysis, and rbsurv analysis), we identified an apoptosis-related gene signature, and further validated its efficiency in the ICGC dataset. Our signature can efficiently stratify risk patients' overall survival in the TCGA dataset and ICGC dataset, suggesting the signature's stability and reliability. Moreover, the signature's risk score presented a significant increase in stage and grade, further confirmed robustness of our signature. By performing univariate cox regression analysis and multivariate cox regression analysis on the clinical traits and signature risk score, we demonstrated that the signature could be served as an independent prognostic signature for the survival of ccRCC. We further constructed a nomogram based on the signature risk score and significant risk factors. The calibration plot for the survival probability indicated a good concordance on the 1-, 3-, and 5-years overall survival in the TCGA dataset. Our GSEA results for the gene signature revealed that many pathways were significantly enriched, of which most were cancer-related pathways. Interestingly, all the significant pathways were enriched in the low-risk group, and none of the significant pathways existed in the high-risk group. From this point of view, the low-risk group might benefit more from the cancer-related pathway. Together, these results provided a potential direction to reveal the underlying mechanism of ccRCC.

Cumulative researchers have discovered that cytotoxic T lymphocytes (CTL, CD8 T cells) and natural killer cells can induce apoptosis by releasing pro-apoptotic mediators from cytotoxic particles, thereby defend against intracellular pathogens and tumors 18. Our study evaluated the relationship between signature risk score and 22 immune cell infiltration level calculated by CIBERSORTx tools. We found that the risk score was positively correlated with CD4 T cells memory activated CD8 T cells. Considering CD8 T cells play an essential role in apoptosis, it's no surprise that CD4 T cells memory activated CD8 T cells were tightly correlated with gene signature. Also, we discovered that the risk score was presented a negative correlation with the pathways, including positive regulation of T cell-mediated immune response to the tumor cell and regulation of T cell-mediated cytotoxicity directed against tumor cell target. Some relevant studies on this point have suggested that apoptosis can promote T-cell-mediated tumor cell destruction 19, 20. Besides, tumors also can evade immune recognition and destruction via the induction of apoptosis in activated T lymphocytes 20. In short, apoptosis is closely associated with T cells' regulation in ccRCC, and this can't be ignored in the immunotherapy of ccRCC.

Although numerous ccRCC potential subtypes based on gene expression have been proposed in recent years, there is no current consensus about molecular taxonomy. To distinguish reliable molecular subtypes of ccRCC, we employed the apoptosis-genes to establish stable molecular subtypes of ccRCC. Three subtypes of ccRCC named C1, C2, and C3 were identified. The subtype C1 was corresponding to a worse survival outcome, while subtype C2 and C3 associated with a better survival outcome. Moreover, previous studies have shown that sorafenib and sunitinib are widely used to treat metastatic RCC patients. Thus, we evaluated the sensitivity of these two drugs by using the GDSC database and the result showed that subtype C1 was more sensitive to the drugs compared to subtype C2 and C3, indicating that the patients in C1 may have more benefit from the two chemo drugs. In addition to drug sensitivity, we also focus on the likelihood of three subtypes responding to immunotherapy. We discovered that subtype C2 and C3 were more likely to respond to immunotherapy than subtype C1, suggesting that the C2 and C3 patients have more likelihood to be responded to the immunotherapy. These results may also partially explain why C2 and C3 may overall have a better prognosis.

Briefly, we sought here to understand the relationship between apoptosis-related genes expression and ccRCC systematically. We constructed a gene signature that has some clinical significance to the prognosis of ccRCC patients. We also identified three molecular subtypes of ccRCC based on the gene expression, which may benefit the treatment of ccRCC patients. However, several limitation need to be acknowledged. First, the sample size in the ICGC dataset was small, lacking some essential clinical information (stage, grade), which limited the downstream analysis. Secondly, there is a lack of some experiments to validate and support our findings.

In summary, we developed and validated an apoptosis-related gene signature of ccRCC. This model can be served as an independent prognostic factor and have some essential functions and clinical significance, which may contribute to understanding the underlying molecular mechanism of ccRCC. To the best of our knowledge, this is the first attempt to make a comprehensively investigation of the prognostic value of the apoptosis-related genes in ccRCC. Besides, we also identified three robust and reliable molecular subtypes. Those patients in subtype C2 and C3 could benefit from immunotherapy and drugs, which may provide an essential reference for clinicians to develop a personalized treatment.

## Supplementary Material

Supplementary figures and tables.

## Figures and Tables

**Table 1:** The clinical information of the 512 ccRCC patients in the TCGA dataset.

Characteristic		Alive (N =343 )	Dead (N =169 )	Total (N=512)	P value
Age	<65	237	89	326	0.0004
	>=65	106	80	186	
Stage	Stage I	214	42	256	<0.0001
	Stage II	43	13	56	
	Stage III	70	48	118	
	Stage IV	16	66	82	
T	T1	213	47	260	<0.0001
	T2	47	21	68	
	T3	82	91	173	
	T4	1	10	11	
M	M0	303	103	406	<0.0001
	M1	15	63	78	
	MX	25	3	28	
N	N0	146	83	229	0.0034
	N1	5	10	15	
	NX	192	76	268	
Gender	Female	115	61	176	0.634
	Male	228	108	336	
Grade	G1	11	0	11	<0.0001
	G2	176	43	219	
	G3	131	72	203	
	G4	20	53	73	
	GX	5	1	6	
Smoking	1-year	161	104	265	0.0059
	2-year	20	5	25	
	3-year	131	52	183	
	4-year	23	3	26	
	5-year	8	5	13	
Radiation	Yes	4	1	5	0.8857
	No	339	168	507	
Pharmaceutical	Yes	11	62	73	<0.0001
	No	332	107	439	

**Table 2:** The clinical information of the 90 ccRCC patients in ICGC dataset.

Characteristic		Alive (N= 29)	Dead (N=61)	Total (N=90)	P value
Age	<65	15	41	56	0.2365
	>=65	14	20	34	
T	T1	10	44	54	0.0072
	T2	6	7	13	
	T3	12	9	21	
	T4	1	1	2	
M	M0	22	59	81	0.0018
	M1	7	1	8	
	MX	0	1	1	
N	N0	24	54	78	0.7255
	N1	1	1	2	
	NX	4	6	10	
Gender	Female	13	26	39	1.000
	Male	16	35	51	

## Abbreviations

ccRCC: Clear cell renal cell carcinoma
ChRCC: Chromophobe renal cell carcinoma
DFS: Disease free survival
GSEA: Gene set enrichment analysis
GSVA: Gene Set Variation Analysis
GDSC: Genomics of Drug Sensitivity in Cancer
ICGC: International Cancer Genome Consortium
IC_50_: Half-maximal inhibitory concentration
K-M: Kaplan-meier
PRCC: Papillary renal cell carcinoma
PCA: Principal components analysis
RCC: Renal cell carcinoma
Rbsurv: Robust likelihood-based survival
ROC: Receiver operating characteristic
SubMap: Subclass mapping
TCGA: The Cancer Genome Atlas
TIDE: Tumor Immune Dysfunction and Exclusion
