# Supplementary material for: Identification of an apoptosis-related prognostic gene signature and molecular subtypes of clear cell renal cell carcinoma (ccRCC)
Source: J Cancer. 2021 Apr 2;12(11):3265–76. doi: 10.7150/jca.51812 (PMC8100811; doi:10.7150/jca.51812)
Supplement: Supplementary file 1 — Supplementary figures and tables. [file jcav12p3265s1.pdf]

Supplementary Material

Figure S1

Risk model validation in the DFS cohort. (A) K-M survival curve analysis of the apoptosis-related gene signature. (B) ROC analysis was performed to evaluate the accuracy of the gene signature. Univariate cox regression and multivariate cox regression analysis to validate the independence of the gene signature

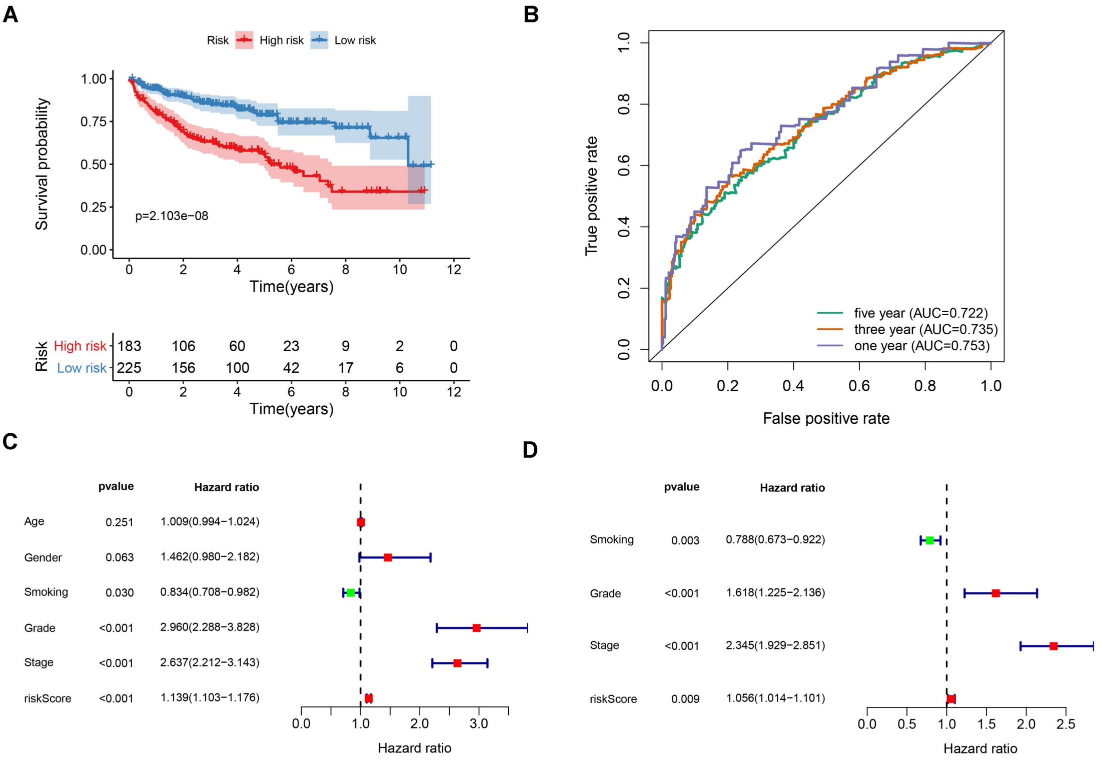

Figure S2

The apoptosis-related gene signature risk score distribution in clinical traits including Stage (A), Grade (B) and Age (C).

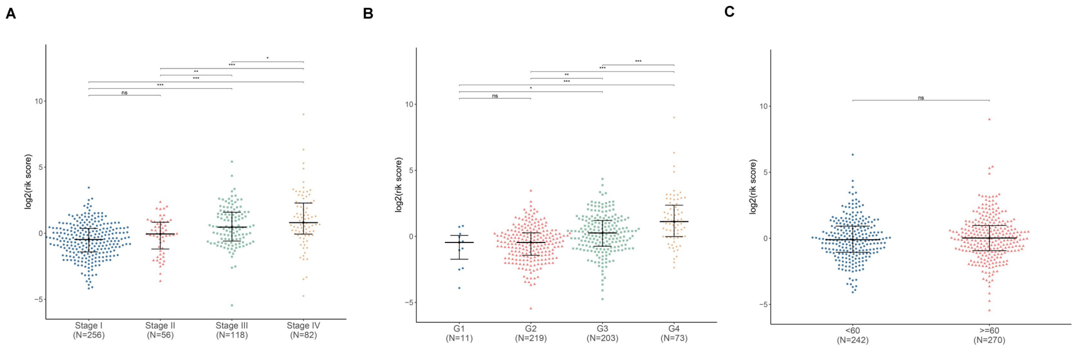

Figure S3

Correlation analysis for the apoptosis-related gene signature risk score and 22

immune cells.

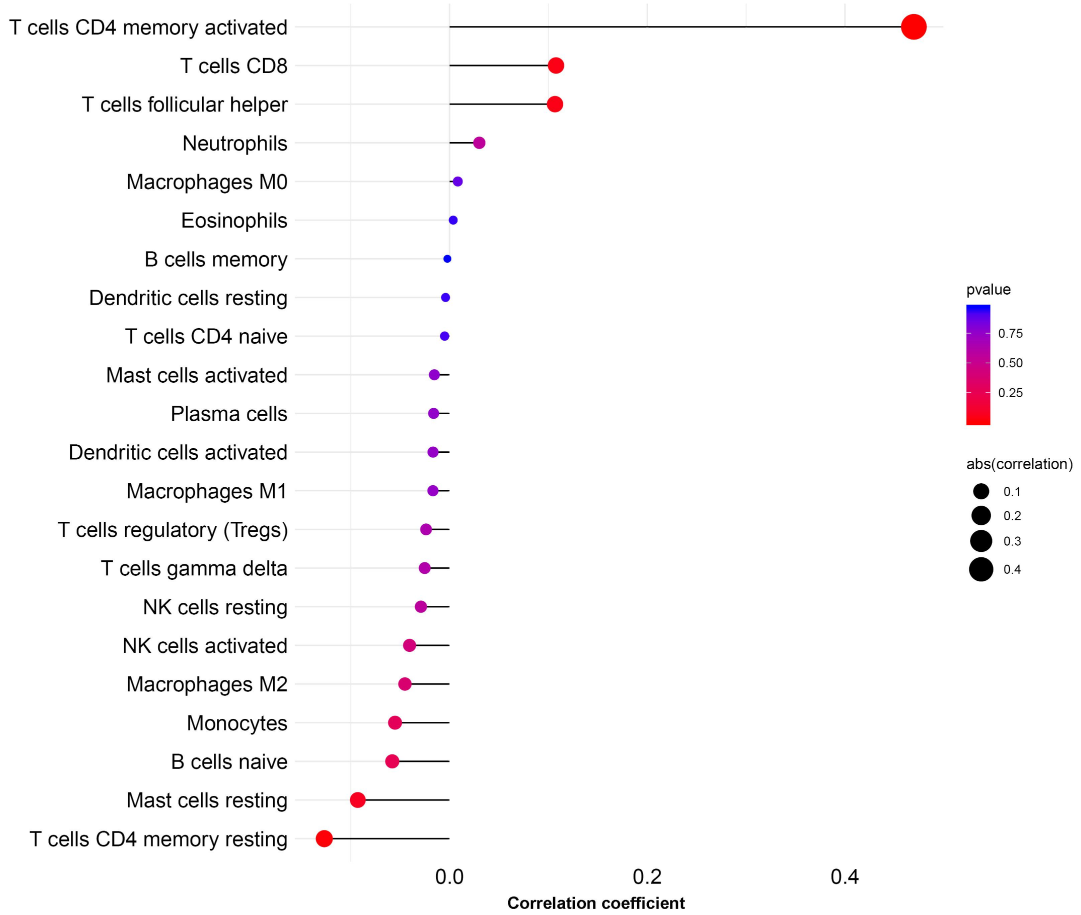

Table S1

20 apoptosis-related genes that selected by multivariate stepwise Cox regression analysis

Gene	coef	HR	HR.95L	HR.95H	P-value
SAT1	-0.004208894	0.995799951	0.992866258	0.998742313	0.005174372
ERBB3	-0.010407818	0.989646156	0.977564405	1.001877225	0.096772062
SLC20A1	0.043199038	1.044145699	1.014182946	1.074993663	0.003637476
CASP9	0.317166259	1.373230865	1.195224123	1.57774845	7.55E-06
PLCB2	0.077917885	1.081033886	1.003168607	1.164943017	0.041061392
ISG20	-0.136136834	0.872723206	0.768782191	0.990717271	0.035369183
GNA15	-0.078508452	0.924494246	0.836858745	1.021306901	0.122334563
DNAJA1	-0.011803034	0.988266348	0.978146232	0.99849117	0.024608961
BTG2	-0.005196107	0.994817369	0.988163812	1.001515727	0.129113568
ADD1	0.033563474	1.034133082	0.988819341	1.081523375	0.142065621
TOP2A	0.058268578	1.05999965	1.023361941	1.097949037	0.001167455
PTK2	-0.064170158	0.937845404	0.861315727	1.021174899	0.139540049
CASP4	0.058218279	1.059946335	0.994990483	1.129142692	0.071181558

BCL2L2	0.04137582	1.042243728	0.990880797	1.096269089	0.108565844
CD44	0.006781168	1.006804213	1.001454449	1.012182555	0.012608751
BID	0.097989501	1.102951205	1.02473679	1.18713544	0.009025223
DFFA	-0.266418455	0.766118474	0.673131238	0.871951091	5.45E-05
GADD45A	-0.01675594	0.98338366	0.970834027	0.996095518	0.010559332
TIMP3	-0.08330072	0.920074421	0.840204335	1.007536982	0.07219265
IFITM3	0.001449026	1.001450076	1.000297014	1.002604467	0.013694003
